# Biological Activities of *Leonotis ocymifolia* (Burm.f.) and Its Antibacterial Activities Against ESKAPE Pathogens

**DOI:** 10.3390/antibiotics14030238

**Published:** 2025-02-26

**Authors:** Tshepo Divine Matlou, Mashilo Mash Matotoka, Talita Jessica Mnisi, Peter Masoko

**Affiliations:** Department of Biochemistry, Microbiology and Biotechnology, University of Limpopo, Private Bag X1106, Sovena 0727, South Africa; tshepodivinematlou08@gmail.com (T.D.M.); mashilo.matotoka@ul.ac.za (M.M.M.); talitajessica912@gmail.com (T.J.M.)

**Keywords:** *Leonotis ocymifolia*, antioxidant, antibacterial, antibiofilm, anti-virulence, ESKAPE pathogens

## Abstract

**Background/Objectives:** The rise in antibiotic-resistant ESKAPE pathogens, which are responsible for severe and hard-to-treat infections, highlights the urgent need for alternative therapeutic agents. While species in the *Leonotis* genus have demonstrated antimicrobial potential, limited research exists on *Leonotis ocymifolia*. This study evaluated the phytochemical profiles and antioxidant, antibacterial, and antibiofilm activities of *L. ocymifolia* leaf and stem extracts. **Methods:** Acidified acetone and hexane were used for extraction, followed by liquid–liquid fractionation with dichloromethane (DCM), ethyl acetate, and butanol. Phytochemicals were profiled using thin-layer chromatography (TLC), while polyphenolic content and antioxidant activity were determined using colorimetric and DPPH assays, respectively. Antibacterial activity was assessed via bioautography and micro-broth dilution assays. Antibiofilm activities were evaluated using crystal violet staining, and metabolic activity was assessed using tetrazolium salt as a cell viability indicator. **Results:** Ethyl acetate fractions had the highest phenolic (98.15 ± 9.63 mg GAE/g) and tannin contents (108.28 ± 8.78 mg GAE/g), with strong DPPH scavenging activity (79–90% at 250 µg/mL). DCM extracts had potent antibacterial activity, with a minimum inhibitory concentration (MIC) of 0.31–0.625 mg/mL against *Pseudomonas aeruginosa*, *Escherichia coli*, and *Klebsiella pneumoniae*. Antibiofilm assays revealed over 50% inhibition across biofilm formation phases, with DCM leaf extracts disrupting biofilms by inhibiting microbial metabolism. **Conclusions:** This study highlights *L. ocymifolia* as a promising source of bioactive compounds with significant antioxidant and antibacterial properties. The DCM and ethyl acetate extracts demonstrated high polyphenol content and effective biofilm inhibition. Further studies are warranted to isolate bioactive compounds and elucidate their mechanisms of action.

## 1. Introduction

The rapid rise in antibiotic resistance among bacterial pathogens represents an urgent global health crisis, undermining decades of medical progress and threatening effective treatments for infectious diseases [1]. Central to this issue are the ESKAPE pathogens. The term “ESKAPE” was coined to highlight the ability of these pathogens—*Enterococcus faecium*, *Staphylococcus aureus*, *Klebsiella pneumoniae*, *Acinetobacter baumannii*, *Pseudomonas aeruginosa*, and *Enterobacter species*—to “escape” the effects of antimicrobial drugs, making them major contributors to the global antibiotic resistance crisis [2]. These bacteria are notorious for their intrinsic resistance mechanisms, including biofilm formation, efflux pump overexpression, and the acquisition of resistance genes through horizontal gene transfer, which enable them to evade the effects of conventional antibiotics. Consequently, they are the primary culprits behind nosocomial infections worldwide, leading to increased morbidity, mortality, and healthcare costs [3,4].

The misuse and overuse of antibiotics in clinical and agricultural settings have further accelerated the development of resistance in these pathogens. For instance, *E. faecium* is a leading cause of vancomycin-resistant enterococcal infections, while *S. aureus*, particularly methicillin-resistant *S. aureus* (MRSA), is associated with severe skin infections, sepsis, and pneumonia [5,6]. Similarly, *K. pneumoniae* produces ESBLs and is implicated in meningitis and pneumonia, while *A. baumannii* and *P. aeruginosa* are notorious for bloodstream and wound infections, especially in burn victims and cystic fibrosis patients [7,8]. These challenges underscore the urgent need for alternative strategies to combat antibiotic resistance.

Medicinal plants, revered for their extensive use in traditional medicine, have emerged as promising sources of bioactive compounds with therapeutic potential. They provide a sustainable reservoir of diverse secondary metabolites that can serve as templates for novel antimicrobial agents [9]. The genus *Leonotis*, known for its ethnomedicinal applications, includes species such as *Leonotis leonurus* and *Leonotis nepetifolia*, which are well-documented for their anti-inflammatory, analgesic, and antimicrobial properties [10]. However, *Leonotis ocymifolia*, despite its widespread traditional use in East and Southern Africa, remains largely understudied.

The limited research on *L. ocymifolia* leaves a significant gap in understanding its phytochemical composition and bioactivities, particularly its potential antibacterial and antibiofilm properties. This study aims to address this gap by investigating the phytochemical profiles, antioxidant activity, and antibacterial efficacy of *L. ocymifolia* leaf and stem extracts against selected ESKAPE pathogens. By exploring its bioactive potential, this research seeks to validate the traditional uses of *L. ocymifolia* and contribute to the development of plant-based strategies to combat antibiotic-resistant infections.

## 2. Results

### 2.1. Phytochemical Screening and Analysis

**Figure 1:**
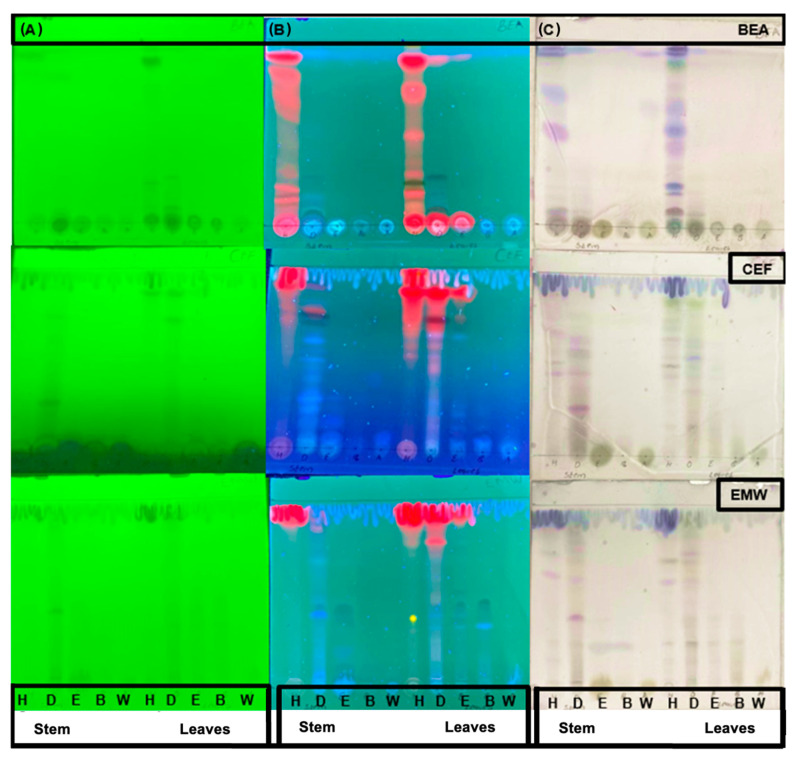
Phytochemical TLC fingerprints of *L. ocymifolia* extracts visualized using UV light at 254 nm (**A**), 365 nm (**B**) and vanillin-sulphuric acid spray (**C**). Key: H—hexane, D—dichloromethane, E—ethyl acetate, B—butanol, and W—water; EMW—ethyl acetate, methanol, water, CEF—chloroform, ethyl acetate, formic acid; and BEA—benzene, ethanol ammonium hydroxide.

The initial phytochemical analysis indicated that both plant parts contained saponins, phlobatannins, tannins, terpenoids, steroids, cardiac glycosides, and flavonoids, while alkaloids were absent (Table 1). Thin-layer chromatography was utilized to generate phytochemical fingerprint profiles of the different extracts (Figure 1). The non-fluorescent compounds were visualized by spraying the plates with vanillin-sulfuric acid (Figure 1C). The chromatograms reveal that the polar (EMW) and intermediate polar (CEF) solvent systems yielded the highest number of bands in the DCM, ethyl acetate, and butanol fractions suggesting a possible aggregation of polyphenols in these solvents as a result of liquid–liquid fractionation. Correspondingly, the non-polar mobile system (BEA) enabled the separation of non-polar compounds mainly in the hexane extracts.

### 2.2. Quantification of Polyphenols

The phenolic, flavonoid, and tannin constituents in the leaf and stem extracts of *L. ocymifolia* were quantified using colorimetric assays, and the findings are presented in Table 2 and Table 3. The ethyl acetate extract exhibited the highest total phenolic content for both the leaves (98.15 ± 9.63 mg GAE/g) and stems (42.73 ± 2.96 mg GAE/g). The DCM extract demonstrated the highest flavonoid content, with values of 200.50 ± 6.65 mg QE/g in the leaves and 54.65 ± 7.03 mg QE/g in the stem. In terms of tannin content, the ethyl acetate extract was predominant from the leaves, with a total tannin content of 108.28 ± 8.78 mg GAE/g, while the DCM extract recorded the highest tannin content in the stem (21.91 ± 0.31 mg GAE/g). Interestingly, the residual water extract showed a negative flavonoid content, indicating a lack of detectable flavonoids under the assay conditions used.

### 2.3. Antioxidant Activity of Extracts

**Figure 2:**
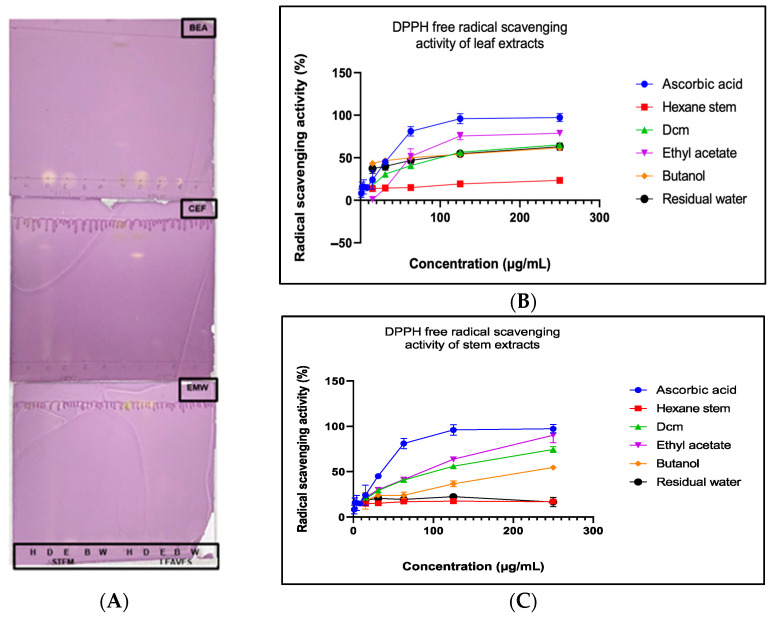
Chromatograms of *L. ocymifolia* extracts developed in BEA, CEF, and EMW mobile phases and sprayed with 0.2% DPPH solution (**A**) and quantitative free radical scavenging activity of leaf (**B**) and stem (**C**) extracts. BEA: benzene, ethanol, ammonia, CEF: chloroform, ethyl acetate, formic acid, EMW: ethyl acetate, methanol, water, H: hexane, D: dichloromethane (DCM), E: ethyl acetate, B: butanol, and W: water.

The antioxidant potential of *L. ocymifolia* stem and leaf extracts was evaluated qualitatively and quantitatively. The TLC-based DPPH assay indicated antioxidant activity, evidenced by slight yellow discoloration against a purple background (Figure 2A). While the extracts exhibited some antioxidant effects, the faint bands suggested that the individual compounds likely had weak antioxidant properties. In the quantitative free radical scavenging activity assay, the extracts demonstrated concentration-dependent antioxidant activity (Figure 2B,C). In the leaves, the butanol fraction exhibited the highest antioxidant activity (EC_50_, 79.74 ± 1.60 µg/mL), whereas in the stem extract, the ethyl acetate fraction showed the strongest antioxidant potential (EC_50_, 96.12 ± 2.67 µg/mL) (Table 4).

### 2.4. Antibacterial Activity of Plant Extracts

The minimum inhibitory concentration (MIC) is the lowest concentration (mg/mL) of an antimicrobial agent that prevents the visible growth of a microorganism after incubation. The DCM leaf extract generally had better broad-spectrum antibacterial activity with an MIC value of 0.63 mg/mL against *P. aeruginosa*, *E. coli*, *E. faecalis*, and *S. aureus* (Table 5). The DCM stem extract exhibited the most potent antibacterial activity, particularly against *P. aeruginosa* and *E. coli* (Gram-negative), as well as *E. faecalis* and *S. aureus* (Gram-positive), with MIC values of 0.31 mg/mL (Table 6). In contrast, the residual water extract from stems displayed minimal activity, with MIC values exceeding 2.5 mg/mL, indicating significantly weaker antibacterial potential. Total antibacterial activity (TA) is defined as the total volume of extract (mL) required to inhibit microbial growth per gram of dried plant material [11]. It is influenced by the extraction yield (mg) per gram of plant material and the minimum inhibitory concentration (MIC), expressed in milliliters per gram (mL/g). The ethyl acetate extract demonstrated superior TA values, with 49.6 mL/g against *K. pneumoniae* and 24.8 mL/g against *P. aeruginosa*, *E. coli*, *E. faecalis*, and *S. aureus.* Similarly, the butanol extract exhibited the highest TA recorded, reaching 161.6 mL/g against *K. pneumoniae*. These findings highlight the potential of specific plant extracts as sources of bioactive compounds for combating resistant bacterial pathogens.

### 2.5. Antibiofilm Activity

We selected plant extracts for antibiofilm evaluation based on their MIC values being (≤0.63 mg/mL). Extracts with lower MIC values are more likely to inhibit biofilm formation, as demonstrated in previous studies [12,13]. This approach allowed us to focus on the most promising candidates for further antibiofilm testing, using assays such as crystal violet staining to assess their ability to disrupt biofilms.

#### 2.5.1. Prevention of Biofilm Formation

**Figure 3:**
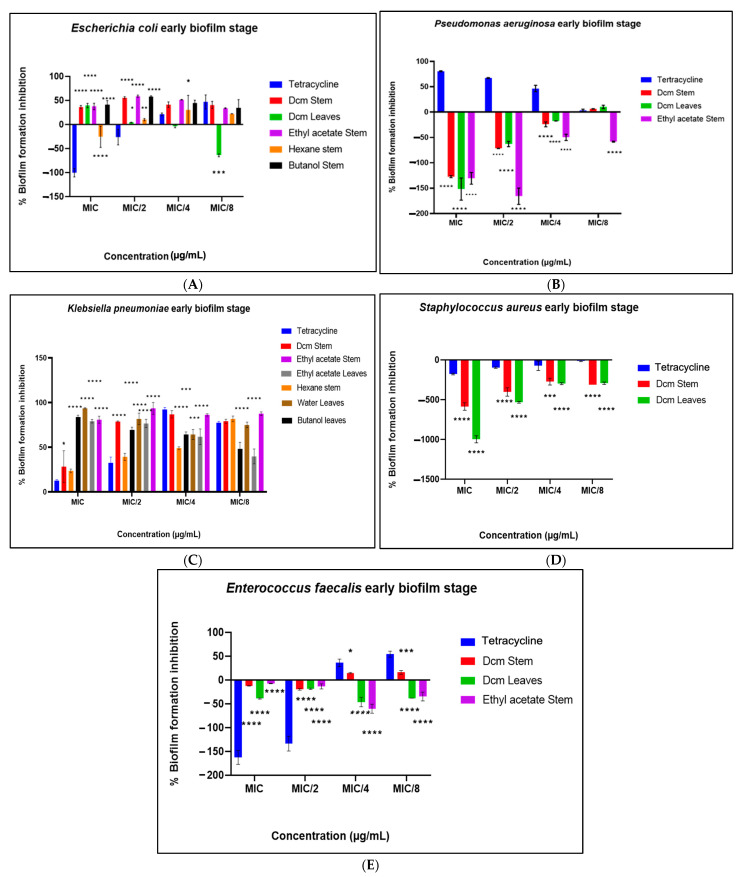
Prevention of biofilm formation against *E. coli* (**A**), *P. aeruginosa* (**B**), *K. pneumoniae* (**C**), *S. aureus* (**D**), and *E. faecalis* (**E**). Data are presented as the mean ± standard deviation of duplicate experiments. One-way ANOVA coupled with Dunnett’s multiple comparisons test was used. A significant difference was observed when (*): *p* < 0.05, (**): *p* < 0.01, (***): *p* < 0.001, and (****): *p* < 0.0001.

The antibiofilm potential of the extracts was evaluated across three distinct phases: biofilm prevention, prevention of cell attachment, and eradication of preformed biofilms. In the biofilm prevention phase (Figure 3A–E), the extracts displayed varied effects across bacterial strains. Notably, significant inhibition of *K. pneumoniae* biofilm formation was observed, while *P. aeruginosa* biofilm formation was moderately inhibited. However, biofilm formation was enhanced in *S. aureus* and *Escherichia coli*, suggesting a strain-specific response. Moderate inhibition of *E. faecalis* biofilm formation was also noted, though to a lesser extent than that observed for *K. pneumoniae*.

#### 2.5.2. Inhibition of Initial Cell Attachment and Mature Biofilm Biomass by Extracts

**Figure 4:**
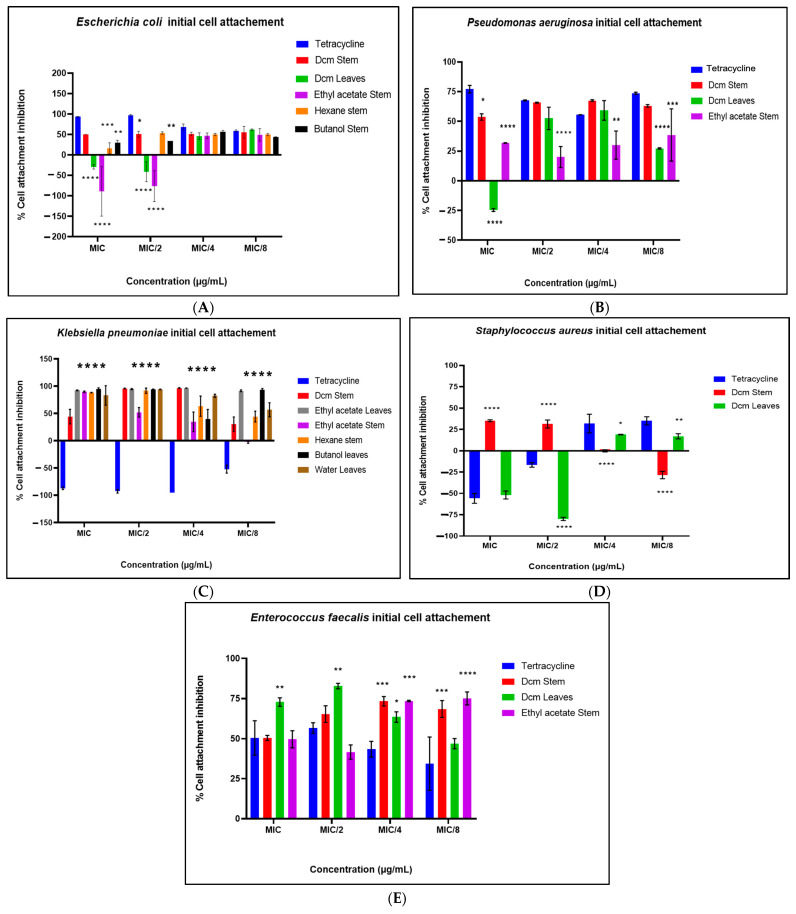
Prevention of cell attachment by the test extracts for *E. coli* (**A**), *P. aeruginosa* (**B**), *K. pneumoniae* (**C**), *S. aureus* (**D**), and *E. faecalis* (**E**). Data are presented as the mean ± standard deviation of duplicate experiments. One-way ANOVA coupled with Dunnett’s multiple comparisons test was used. A significant difference was observed when (*): *p* < 0.05, (**): *p* < 0.01, (***): *p* < 0.001, and (****): *p* < 0.0001.

**Figure 5:**
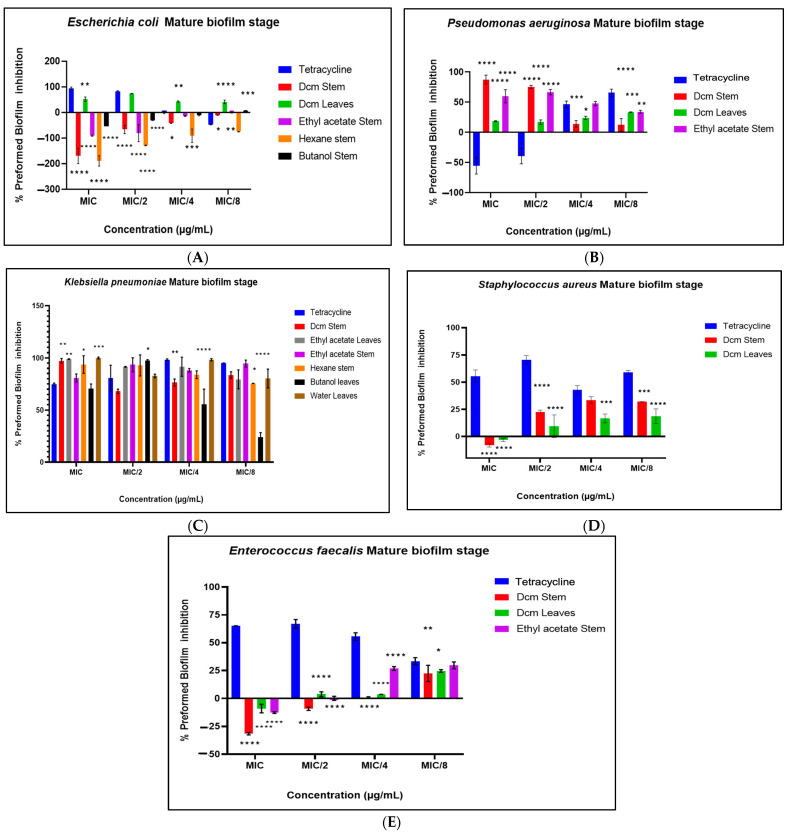
Eradication of preformed biofilm by the test extracts against *E. coli* (**A**), *P. aeruginosa* (**B**), *K. pneumoniae* (**C**), *S. aureus* (**D**), and *E. faecalis* (**E**). Data are presented as the mean ± standard deviation of duplicate experiments. One-way ANOVA coupled with Dunnett’s multiple comparisons test was used. A significant difference was observed when (*): *p* < 0.05, (**): *p* < 0.01, (***): *p* < 0.001, and (****): *p* < 0.0001.

During the inhibition of the cell attachment phase (Figure 4A–E), the extracts demonstrated substantial inhibition of biofilm formation in *E. faecalis*, *P. aeruginosa*, and *K. pneumoniae*, with inhibition percentages exceeding 60% in certain cases. Conversely, an enhancement of biofilm formation was again noted for *S. aureus* and *E. coli*, reinforcing the differential impact of the extracts on these strains. In the eradication of the preformed biofilms phase (Figure 5A–E), the extracts exhibited strong antibiofilm activity against mature biofilms of *K. pneumoniae* and *P. aeruginosa*. Extracts such as the ethyl acetate stem and dichloromethane (DCM) stem extracts demonstrated broad-spectrum antibiofilm activity, achieving over 60% inhibition across multiple strains, including *P. aeruginosa*, *K. pneumoniae*, and *E. faecalis*. Interestingly, preformed biofilms of *E. coli* were enhanced during this phase, highlighting the complexity and strain-specific effects of the extracts. These findings suggest that the tested plant extracts exhibit significant antibiofilm activity, particularly against Gram-negative pathogens, with varying efficacy across different biofilm phases.

### 2.6. Metabolic Activity of Biofilms

**Figure 6:**
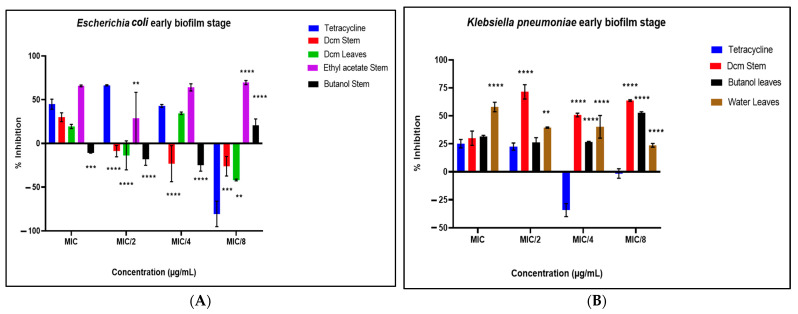
The effect of selected sub-MIC concentrations of extracts on the metabolic activity of *E. coli* (**A**) and *K. pneumoniae* (**B**) during the early biofilm-forming phase. Results are presented as mean ± standard deviation of duplicate experiments. One-way ANOVA coupled with Dunnett’s multiple comparisons test was used. A significant difference was observed when (**): *p* < 0.01, (***): *p* < 0.001, and (****): *p* < 0.0001.

The metabolic activity of bacterial biofilms was evaluated during two key stages: biofilm formation prevention and the eradication of established biofilms. In the prevention stage (Figure 6A,B), the ethyl acetate extract demonstrated significant inhibitory effects against *E. coli*, achieving over 50% inhibition across all tested sub-MIC concentrations. Similarly, all extracts exhibited inhibitory activity against the formation and growth of *K. pneumoniae* biofilms, with the DCM extract showing notable inhibition at MIC/2, MIC/4, and MIC/8 concentrations, while the butanol leaf extract was particularly effective at MIC/8.

**Figure 7:**
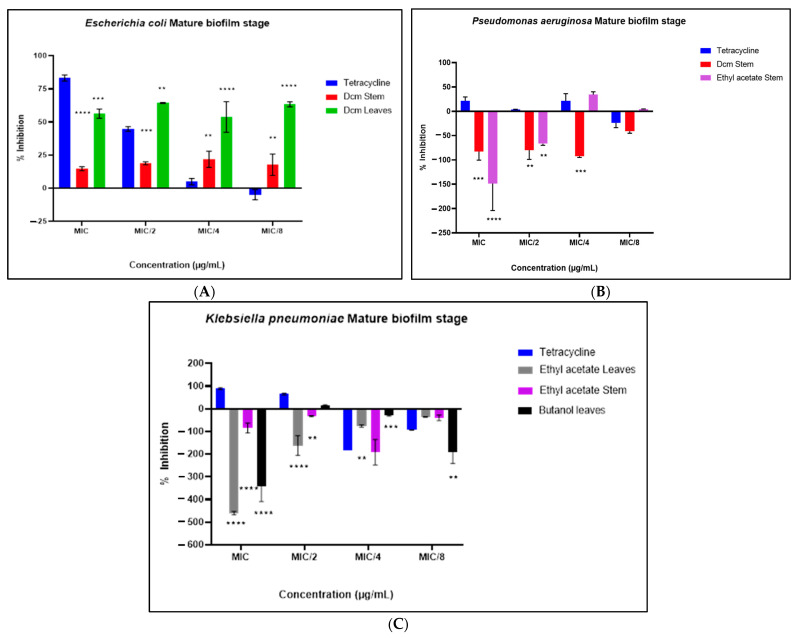
The effect of selected sub-MIC concentrations of extracts on the metabolic activity of *E. coli* (**A**), *P. aeruginosa* (**B**), and *K. pneumoniae* (**C**) during the eradication of preformed biofilms. Results are presented as mean ± standard deviation of duplicate experiments. One-way ANOVA coupled with Dunnett’s multiple comparisons test was used. A significant difference was observed when (**): *p* < 0.01, (***): *p* < 0.001, and (****): *p* < 0.0001.

During the eradication phase of preformed biofilms, the metabolic activity of treated mature biofilms (suggested that the observed disruption in biofilm biomass from earlier assays (eradication of biofilm biomass) may not directly correlate with metabolic inhibition in regards to *K. pneumoniae* and *P. aeruginosa* (Figure 7B,C). However, the metabolic activity of *E. coli* was significantly reduced, with the DCM leaf extract achieving over 50% inhibition across all sub-MIC concentrations, emphasizing its strong potential for metabolic disruption against *E. coli*.

## 3. Discussion

The rapid emergence of drug-resistant bacteria, particularly biofilm-forming pathogens, has created an urgent need for alternative antimicrobial strategies. This study investigates the potential of *L. ocymifolia* extracts as anti-virulence agents, focusing on their antibiofilm and metabolic activity, while also exploring antioxidant and antibacterial properties. The findings demonstrate a promising link between these properties, highlighting the potential of *L. ocymifolia* in addressing antimicrobial resistance.

The antioxidant activities of the extracts, particularly those derived from dichloromethane ethyl acetate and butanol, were attributed to their high polyphenol and flavonoid content. Previous studies have also established a correlation between high antioxidant activity and elevated polyphenol content in various plant species [14,15]. A study by Mufti et al. [16] suggested that some therapeutic properties of *L. ocymifolia* extracts could be related to their antioxidant and anti-inflammatory activities. In a separate study on a *Leonotis* species by Tonisi et al. [17], *L. leonurus* exhibited high phenolic and flavonoid contents, which correlated with significant radical-scavenging effects against 2,2-diphenyl-1-picrylhydrazyl (DPPH), 2,2′-azinobis-(3-ethylbenzothiazoline-6-sulfonate) (ABTS·+), hydrogen peroxide, and nitric oxide, along with notable metal-chelating activity. These findings further support the association between antioxidant activity and members of the *Leonotis* genus. These antioxidants play a critical role in mitigating oxidative stress within bacterial cells, which is essential for biofilm integrity and bacterial survival. By disrupting redox balance, antioxidants may potentiate the antibacterial efficacy of the extracts, creating a synergistic effect against biofilm-forming pathogens.

The antibacterial efficacy of the extracts varied across bacterial strains, with notable activity against *P. aeruginosa* and *E. coli*. The dichloromethane stem extract exhibited strong antibacterial properties, with MIC values as low as 0.31 mg/mL. The differences in activity between extracts underscore the role of solvent polarity in extracting bioactive compounds. Ethyl acetate and butanol extracts demonstrated high total activity values, suggesting their potential for therapeutic applications due to their ability to retain antibacterial effectiveness even at significant dilutions. A recent study by Oyedeji-Amusa et al. [18] demonstrated that the ethyl acetate and butanol leaf fractions derived from DCM/methanol crude extracts exhibited notable antibacterial activity against *E. coli* (ATCC 8739), *S. aureus* (ATCC 25923), and *K. pneumoniae* (ATCC 13883), with MIC values ranging from 1 to 0.25 mg/mL. In this study, tetracycline used as a positive control demonstrated notable broad-spectrum antibacterial activity against all test ATCC pathogens where MIC values ranged from 0.625 mg/mL to 0.156 mg/mL. In a separate study, tetracycline exhibited MIC values of 0.031 µg/mL against *E. faecalis* and 0.063 µg/mL against *E. coli* O175:H7 [19]. Additionally, an MIC of 0.78 µg/mL was recorded against *S. aureus* [20]. These findings suggest that the antibacterial activity of tetracycline varies depending on the strain tested. However, in the present study, the fractions demonstrated comparable antibacterial efficacy.

The hexane extracts exhibited moderate activity in this study. However, previous research by Oyedeji et al. [21] demonstrated that the essential oils of *L. ocymifolia* possessed significant antibacterial activity against *Bacillus cereus*, *Micrococcus kristinae*, *Staphylococcus epidermidis*, *S. aureus*, *E. coli*, *P. aeruginosa*, and *Shigella sonnei*, with MIC values ranging from 1.25 to 0.156 mg/mL. Screening antibacterial activity using non-resistant bacterial strains helps establish a compound’s baseline efficacy before testing against resistant strains. These strains facilitate comparisons with standard antibiotics and simplify mechanism studies, as they lack resistance factors like efflux pumps.

Biofilm formation is a key virulence factor for pathogens, especially ESKAPE organisms, as it enhances antibiotic resistance. This study assessed the extracts’ capacity to inhibit biofilm formation, disrupt biofilm development, and eradicate pre-formed biofilms. Relying on MIC in this study was crucial since tetracyclines are bacteriostatic rather than bactericidal [19]; thus, minimum bactericidal concentrations (MBCs) were not examined further. More importantly, when evaluating antibiofilm or anti-virulence properties, bactericidal effects are less desirable, as maintaining microbial viability is essential for studying these characteristics [22].

Noteworthy antibiofilm activity was observed, with *K. pneumoniae* showing significant susceptibility at all stages of biofilm development. The dichloromethane stem extract was particularly effective, achieving up to 98.34% inhibition of pre-formed biofilms at sub-MIC concentrations. Sub-MIC evaluations revealed that the extracts influence biofilm formation without causing complete bacterial lysis, simulating clinically relevant scenarios [23]. This approach is critical for understanding how targeting biofilm structure and bacterial signalling pathways can improve susceptibility to conventional antibiotics [24]. In certain bacterial pathogens, tetracycline was observed to promote biofilm formation at various concentrations (Figure 3, Figure 4 and Figure 5). This phenomenon is not new, as previous studies have reported tetracycline-induced biofilm enhancement in *S. aureus* [25], *K. pneumoniae* [26], and *Pseudomonas* spp. [26] and *E. coli* [27]. Research has suggested that this increase in biofilm formation in the presence of tetracycline may be associated with elevated levels of cyclic diguanylate (c-di-GMP), a second messenger known to positively regulate biofilm development [28].

Further evidence of the extracts’ efficacy was provided by a reduction in metabolic activity within biofilms, measured using INT. The dichloromethane stem extract significantly inhibited metabolic activity in both *E. coli* and *K. pneumoniae*, with strong correlations to reduce biofilm biomass. For instance, metabolic activity inhibition in *K. pneumoniae* reached 71.43% at sub-MIC levels, highlighting the potential of these extracts to interfere with biofilm-associated physiological processes. These findings are consistent with Sandasi et al. [29], who observed that targeting metabolic activity within biofilms can decrease microbial viability without necessarily causing structural disintegration. This suggests that extracts with potent antioxidant and antibacterial properties may also disrupt metabolic pathways essential for biofilm maintenance and virulence.

## 4. Materials and Methods

### 4.1. Plant Collection and Storage

*L. ocymifolia* was collected in the Autumn of 2024 from the Mankweng area (GPS coordinates: −23.883668 latitude, 29.707878 longitude), Limpopo Province, South Africa. The plants were identified by Dr. Egan Bronwyn, a botanist and the curator of the Larry Leach Herbarium, based at the University of Limpopo. The leaves and stems were separated. These plant materials were subsequently dried at ambient temperature, away from direct sunlight, by placing them on laboratory benches in the dark. Upon drying, an electric grinder (Waring Laboratory Blender LB20) was used to grind the leaves and stems into a fine powder. The powdered plant material was transferred into airtight bottles and stored in a dark container until further use to preserve its stability and prevent any photo-degradation.

### 4.2. Extraction and Fractionation Procedure

The dried, ground leaves and stems of *L. ocymifolia* (20 g) were subjected to simultaneous extraction with hexane and 70% acidified acetone (SupraSolv^®^, Darmstadt, Germany). The extracts were filtered using Whatman No. 1 filter paper, and the filtrates were collected into separate beakers. The 70% acidified acetone crude was sequentially fractionated with solvents of increasing polarity, namely dichloromethane (DCM) (SupraSolv^®^, Darmstadt, Germany), ethyl acetate (SupraSolv^®^, Darmstadt, Germany), and butanol (SupraSolv^®^, Darmstadt, Germany), with each fraction collected in a separate beaker. The residual water phase was retained as the final fraction [30]. The collected extracts were partially evaporated under a fan and transferred into pre-weighed glass vials for drying. The mass of the dried extracts was calculated by subtracting the weight of the empty vials from the final weight of the vials containing the dried residues. The extracts were all reconstituted in acetone (SupraSolv^®^, Darmstadt, Germany) to the desired concentration (10 mg/mL).

### 4.3. Phytochemical Screening and Analysis

Standard phytochemical screening techniques, as described by Borokini and Omotayo [31] with slight modifications, were employed to assess the presence of key bioactive compounds. Following the preliminary screening, the fractions were further analysed and profiled using TLC to separate the phytochemicals based on their chemical properties.

### 4.4. Quantification of Polyphenolics

The total phenolic content (TPC) and total tannin content (TTC) in the *L. ocymifolia* extracts were measured using the Folin–Ciocalteu reagent method, with slight modifications following the protocol described by Tambe and Bhambar [32]. Ten (10) µL of extract (10 mg/mL) was diluted with 490 µL of water, and then 0.25 mL of Folin–Ciocalteu reagent (Sigma Aldrich^®^, St. Louis, MO, USA) was added. After 1.25 mL of sodium carbonate (Supelco^®^, Bellefonte, PA, USA) (7% for TPC and 35% for TTC) was added, the mixtures were incubated in the dark at room temperature for 30 min. Absorbance was measured at 550 nm (TPC) and 725 nm (TTC) using a UV/VIS spectrophotometer (Thermo Scientific, CAT:840-209800, Waltham, MA, USA, Genesys 10S UV-VIS, Menlo Park, CA, USA). The experiment was conducted in triplicate and repeated three times. The TPC was derived from the equation of the gallic acid (Sigma Aldrich^®^, St. Louis, MO, USA) standard curve (y = 0.6918x + 0.0039, R^2^ = 0.9976), while TTC was determined using the equation from the gallic acid standard curve (y = 0.7918x + 0.049, R^2^ = 0.9785). The TPC and TTC were expressed as milligrams of gallic acid equivalents per gram of extract (mg GAE/g extract).

The total flavonoid content in *L. ocymifolia* extracts was measured using the aluminum chloride (Sigma Aldrich^®^, St. Louis, MO, USA) colorimetric assay, with slight modifications based on the method described by Tambe and Bhambar [32]. A 100 µL of 10 mg/mL extracts were mixed with 4.9 mL distilled water. Then, 300 µL of 5% NaNO_2_ (Supelco^®^, Bellefonte, PA, USA) was added and left at 25 °C for 5 min, followed by 300 µL of 10% AlCl_3_ and another 5 min incubation. Afterward, 2 mL of 1 M NaOH (Supelco^®^, Bellefonte, PA, USA) was added, and the volume was adjusted to 10 mL with distilled water. Quercetin (Sigma Aldrich^®^, St. Louis, MO, USA) (500–31.5 µg/mL) served as the standard. Absorbance was measured at 510 nm using a UV/VIS spectrophotometer (Thermo Scientific, CAT:840-209800, Waltham, MA, USA, Genesys 10S UV-VIS, Menlo Park, CA, USA). A blank was prepared with distilled water instead of extracts. Total flavonoid content was expressed as mg QE/g extract. The experiment was conducted in triplicate and repeated three times. The flavonoid content was calculated using the equation from the quercetin standard curve (y = 0.1129x + 0.0043).

### 4.5. Antioxidant Activity Analysis

The qualitative antioxidant properties of the plant extracts were determined using the DPPH (2,2-diphenyl-1-picrylhydrazyl) assay in conjunction with TLC. The free radical scavenging activity of the plant extracts was quantified using the DPPH method, based on the protocol by Chigayo et al. [33]. Different extract concentrations (250–15.63 µg/mL) were prepared in a 1 mL solution. L-ascorbic acid served as the standard and was prepared in the same concentration range. To each 1 mL solution, 2 mL of 0.2 mmol/L DPPH (Sigma Aldrich^®^, St. Louis, MO, USA) (dissolved in methanol, SupraSolv^®^, Darmstadt, Germany) was added and mixed thoroughly using a vortex. The mixtures were then incubated in the dark for 30 min. A control was prepared by mixing 2 mL of 0.2 mmol/L DPPH with 1 mL of distilled water. After incubation, all samples were analysed using a UV/VIS spectrophotometer (Thermo Scientific, CAT:840-209800, Waltham, MA, USA, Genesys 10S UV-VIS, Menlo Park, CA, USA). The EC_50_ values for the DPPH analysis were determined by plotting activity against concentration and determining which concentration would have yielded an activity of 50%.

### 4.6. Antibacterial Activity

The antibacterial activity of the extracts was quantified by determining the minimum inhibitory concentrations (MICs) using a modified broth microdilution method, adapted from Eloff [34]. The stock bacterial cultures were incubated overnight at 37 °C, and working concentrations were adjusted with nutrient broth media (Oxoid Ltd., Basingstoke, UK) such that the appropriate concentrations of *E. coli* (ATCC 25922) (2 × 10^8^ CFU/mL), *S. aureus* (ATCC 25923). (2 × 10^8^ CFU/mL), *E. faecalis* (ATCC 29212) (3 × 10^7^ CFU/mL), *K. pneumoniae* (ATCC 10031) (2 × 10^7^ CFU/mL), and *P. aeruginosa* (ATCC 27852) (3 × 10^7^ CFU/mL) were obtained using UV/VIS spectrophotometer at OD_600_ as per Clinical and Laboratory Standards Institute (CLSI) methodologies. A total of 100 µL of sterile nutrient broth (Oxoid Ltd., Basingstoke, UK) was dispensed into each well of a 96-well microtiter plate, and extracts (10 mg/mL) were serially diluted two-fold with the media within the plate to achieve final concentrations ranging from 2.5 mg/mL to 0.02 mg/mL in a 100 µL volume. Then, 100 µL of the standardized bacterial culture for each species was introduced into the designated wells, and the plates were incubated at 37 °C for 24 h. Following incubation, 40 µL of *p*-iodonitrotetrazolium chloride (INT) (Sigma Aldrich^®^, St. Louis, MO, USA) solution (0.2 mg/mL) was added, followed by a 30 min incubation. Tetracycline (Glentham Life Sciences, Corsham, UK) served as a positive control/standard for the respective bacterial strains, while media only was used as the negative and sterile control, respectively.

### 4.7. Antibiofilm Activity Assays

The antibiofilm activity of the extracts was evaluated using a crystal violet assay to assess biofilm prevention, inhibition of initial attachment, and eradication of preformed biofilms.

#### 4.7.1. Prevention of Biofilm Formation

The test organisms were standardised using a UV/VIS spectrophotometer (Thermo Scientific, CAT:840-209800, Waltham, MA, USA, Genesys 10S UV-VIS, Menlo Park, CA, USA). as described in Section 4.6. For the assay, 100 µL of nutrient broth (Oxoid Ltd., Basingstoke, UK) was added into each well, followed by the addition of 100 µL of each bacterial suspension into the wells. Subsequently, 100 μL extracts were added to bacterial wells such that final concentrations of MIC, 1/2 MIC, 1/4 MIC, and 1/8 MIC were obtained in the treatment. Untreated cultures served as negative controls, while tetracycline (Glentham Life Sciences, Corsham, UK) was used as the standard (positive control) in the assay. The plates were incubated at 37 °C for 24 h [35], after which biofilm formation was evaluated using the crystal violet staining method [36].

#### 4.7.2. Inhibition of Initial Attachment

The test organisms were standardised using a UV/VIS spectrophotometer (Thermo Scientific, CAT:840-209800, Waltham, MA, USA, Genesys 10S UV-VIS, Menlo Park, CA, USA), as described in Section 4.6, and 100 μL of the bacterial suspension was dispensed into flat-bottomed 96-well plates, followed by incubation at 37 °C for 4 h to allow initial cell attachment, without agitation. After this period, 100 μL of the extracts were added to the wells at concentrations corresponding to multiples of their MIC values. The plates were then further incubated at 37 °C for 24 h without agitation. Tetracycline (Glentham^®^ Life Sciences, Corsham, UK) was used as the positive control/standard, while sterile media and untreated culture served as the negative controls [37]. The biomass quantification was performed using the crystal violet staining method.

#### 4.7.3. Eradication of Mature Biofilms

A 100 μL of the test organisms, prepared similarly to Section 4.6, was introduced into flat-bottomed 96-well plates and incubated at 37 °C for 48 h. Following incubation, 100 μL of plant extracts were added to the wells to achieve final concentrations ranging from four times the MIC (4 × MIC) to half the MIC (0.5 × MIC). The plates were then further incubated at 37 °C for 24 h. Tetracycline (Glentham^®^ Life Sciences, Corsham, UK) served as the positive control/standard, while untreated culture and sterile media were used as negative controls [37]. Biofilm biomass eradication was assessed using the crystal violet (CV) staining method.

#### 4.7.4. Crystal Violet Assay

Biofilm biomass was quantified by washing the treatment plates three times with sterile distilled water followed by oven-drying at 60 °C for 45 min. The wells were then stained with 100 μL of 0.1% crystal violet (Glentham^®^ Life Sciences, Corsham, UK) (in methanol, SupraSolv^®^, Darmstadt, Germany) and incubated at room temperature for 15 min. Unabsorbed stains were removed by washing the plates with sterile distilled water and the plates were air-dried. The absorbed crystal violet was solubilised by adding 125 μL of ethanol (SupraSolv^®^, Darmstadt, Germany), and the solution was transferred to a new plate. The absorbance was measured at 590 nm using a microplate reader (Thermo Scientific, CAT:1530, Multiskan Sky, Singapore) [36]. Biofilm inhibition was calculated using the formula:

$$\%\mathrm{inhibition}=(\frac{OD\;control-OD\;experimental}{OD\;control})\times 100$$

### 4.8. Metabolic Activity Screening

The effect of the extract on the metabolic activity of biofilm biomass during both the formation and mature stages was assessed following the method outlined by Mohsenipour and Hassanshahian [38]. Only microorganisms exhibiting more than 50% inhibition in the antibiofilm assay were selected for this analysis, specifically *K. pneumoniae* (2 × 10^7^ CFU/mL) (*E. coli* (2 × 10^8^ CFU/mL) and *P. aeruginosa* (3 × 10^7^ CFU/mL). The preparation of bacterial biofilm treatments followed the procedures described in Section 4.7.1 and Section 4.7.3, where tetracycline (Glentham^®^ Life Sciences, Corsham, UK) was used as a positive control/standard, and untreated culture and sterile media were used as negative controls. After 24 h treatments, non-adherent cells were eliminated by washing the wells with 100 µL of phosphate-buffered saline (PBS) (Hyclone^TM^, Marlborough, MA, USA). PBS was prepared using 8 g sodium chloride (Supelco^®^, Bellefonte, PA, USA), 0.2 g potassium chloride (Supelco^®^, Bellefonte, PA, USA), 1.44 g sodium phosphate dibasic (Supelco^®^, Bellefonte, PA, USA), and 0.245 g potassium phosphate monobasic (Supelco^®^, Bellefonte, PA, USA), with pH adjusted to 7.4. Next, 40 µL of 0.2 mg/mL INT was added to each well and the plates were incubated in the dark at 37 °C for 30 min. Metabolic activity was then measured at 490 nm using a microplate reader (Thermo Scientific, CAT:1530, Multiskan Sky, Singapore). The percentages of reduced biofilm metabolic activity of the treated and untreated biofilms were determined.

### 4.9. Statistical Analysis

All experiments were conducted in triplicate, and the results were presented as the mean ± standard deviation (SD). Statistical analyses were carried out using GraphPad Prism version 9.0, employing one-way ANOVA followed by Tukey’s HSD test to evaluate significant differences between the extracts. Different letters indicate statistically significant differences (*p* < 0.05), while identical letters denote no significant difference (*p* > 0.05).

## 5. Conclusions

The strong antibiofilm and antibacterial properties of *L. ocymifolia* (DCM and ethyl acetate leaf subfractions) highlight its potential in combating biofilm-associated infections, particularly in nosocomial settings, with applications in wound dressings, medical coatings, and oral healthcare products to help prevent persistent infections and reduce antibiotic resistance. Additionally, its antioxidant, antibiofilm, and antibacterial activities make it a valuable natural alternative for food preservation, active packaging, and surface sanitization, contributing to enhanced food safety and extended shelf life by preventing microbial contamination and oxidative degradation. The defatting process with hexane successfully removed non-polar impurities, enhancing the activity of polar compounds (polyphenolics). Future research should aim to isolate specific bioactive compounds, understand their mechanisms of action, and explore their synergistic interactions with existing antibiotics. Bioassay-guided fractionation employing chromatographic techniques can be utilized to isolate bioactive compounds. Analytical methods, such as liquid chromatography–mass spectrometry (LC–MS) and nuclear magnetic resonance (NMR), can further aid in the characterization and structural elucidation of these bioactive molecules. Additionally, in vivo studies are crucial to confirm the clinical relevance of these findings and to assess the safety and efficacy of *L. ocymifolia*-based therapies.

## Figures and Tables

**Table 1:** Phytochemical constituents detected in *L. ocymifolia* stem and leaf extracts.

Phytochemical Constituents	Stem	Leaves
Saponins	+	+
Phlobatannins	+	+
Tannins	+	+
Terpenoids	+	+
Steroids	+	+
Alkaloids	-	-
Flavonoids	+	+
Cardiac glycosides	+	+

**Table 2:** Total phenolic, flavonoid, and tannin contents in leaf extracts of *L. ocymifolia*.

Extraction Solvent	Phenolic Content (mg GAE/g Extract)	Flavonoid Content (mg QE/g Extract)	Tannin Content (mg GAE/g Extract)
Hexane	27.09± 3.91 ^b,c^	31.62 ± 8.77 ^b^	23.48 ± 3.96 ^b^
Dichloromethane	93.68 ± 4.91 ^d^	200,50 ± 6.65 ^e^	103.03 ± 4.16 ^c^
Ethyl acetate	98.15 ± 9.63 ^e^	141.75 ± 10.27 ^d^	108.28 ± 8.78 ^c^
Butanol	27.91 ± 0.82 ^a,b,c^	23.94 ± 1.84 ^a,b^	22.71 ± 5.52 ^a,b^
Residual water	36.73 ± 3.88 ^c,d^	19.81 ± 1.02 ^a,b^	26.18 ± 2.66 ^b^

GAE: gallic acid equivalence; QE: quercetin equivalence. Results are reported as mean ± standard deviation. Different letters represent statistically significant differences (*p* < 0.05) and the same letters are insignificant (*p* > 0.05).

**Table 3:** Total phenolic, flavonoid, and tannin contents in stem extracts of *L. ocymifolia*.

Extraction Solvent	Phenolic Content (mg GAE/g Extract)	Flavonoid Content (mg QE/g Extract)	Tannin Content (mg GAE/g Extract)
Hexane	1.58 ± 1.56 ^a^	13.91 ± 0.89 ^a^	0.66 ± 0.72 ^a^
Dichloromethane	33.75 ± 8.51 ^c,d^	54.65 ± 7.03 ^c^	21.91 ± 0.31 ^a,b^
Ethyl acetate	42.73 ± 2.96 ^d^	34.28 ± 0 ^b,c^	16.78 ± 2.76 ^a,b^
Butanol	13.58 ± 0.33	15.09 ± 2.05	5.89 ± 3.04
Residual water	4.81 ± 1.96 ^a,b^	-	2.62 ± 0.61 ^a,b^

GAE: gallic acid equivalence; QE: quercetin equivalence. Results are reported as mean ± standard deviation. Different letters represent statistically significant differences (*p* < 0.05) and the same letters are insignificant (*p* > 0.05).

**Table 4:** Half maximal effective DPPH scavenging concentrations (EC_50_) of the plant extracts.

Plant Part	Solvent	Free-Radical Scavenging Activity EC_50_ (μg/mL)
Leaves	Hexane	813.57 ± 0.61
	Dichloromethane	137.31± 1.17
	Ethyl acetate	69.48 ± 1.59
	Butanol	79.74 ± 1.60
	Residual water	93.49 ± 1.975
Stem	Hexane	1354.25 ± 1.63
	Dichloromethane	121.94 ± 1.56
	Ethyl acetate	96.12 ± 2.67
	Butanol	208.48 ± 1.23
	Residual water	1562.96 ± 2.91
L-ascorbic acid		35.53 ± 0.53

**Table 5:** Antibacterial activity of *L. ocymifolia* leaf extracts.

Plant Part	Solvent	*K. pneumoniae*		*P. aeruginosa*		*E. coli*		*E. faecalis*		*S. aureus*	
		MIC (mg/mL)	TA (mL/g)	MIC (mg/mL)	TA (mL/g)	MIC (mg/mL)	TA (mL/g)	MIC (mg/mL)	TA (mL/g)	MIC (mg/mL)	TA (mL/g)
Leaves	H	1.25	127.2	-	-	-	-	-	-	1.25	127.2
	DCM	1.25	17.6	0.625	35.2	0.625	35.2	0.625	35.2	0.625	35.2
	EA	0.625	49.6	1.25	24.8	1.25	24.8	1.25	24.8	1.25	24.8
	BUT	0.625	161.6	2.5	80.8	1.25	80.8	2.5	40.4	-	-
	RW	0.625	902.4	-	-	-	-	-	-	-	-
	Tetracycline	0.156		0.625		0.156		0.156		<0.019	

MIC: minimum inhibitory concentration, TA: total activity, H: hexane, DCM: dichloromethane, EA: ethyl acetate, BUT: butanol, RW: residual water extract, and (-) indicates values not determined as they may be greater than 2.5 mg/mL.

**Table 6:** Antibacterial activity of *L. ocymifolia* stem extracts.

Plant Part	Solvent	*K. pneumoniae*		*P. aeruginosa*		*E. coli*		*E. faecalis*		*S. aureus*	
		MIC (mg/mL)	TA (mL/g)	MIC (mg/mL)	TA (mL/g)	MIC (mg/mL)	TA (mL/g)	MIC (mg/mL)	TA (mL/g)	MIC (mg/mL)	TA (mL/g)
Stem	H	0.3125	348.8	1.25	87.2	0.625	161.6	1.25	87.2	-	-
	DCM	0.3125	89.6	0.3125	89.6	0.3125	89.6	0.625	44.8	0.625	44.8
	EA	0.625	35.2	0.3125	70.4	0.3125	70.4	0.3125	70.4	1.25	17.6
	BUT	-	-	2.5	59.6	0.625	236.8	-	-	-	-
	RW	-	-	-	-	-	-	-	-	-	-
	Tetracycline	0.156		0.625		0.156		0.156		<0.019	

MIC: minimum inhibitory concentration, TA: total activity, H: hexane, DCM: dichloromethane, EA: ethyl acetate, BUT: butanol, RW: residual water extract, and (-) indicates values not determined as they may be greater than 2.5 mg/mL.

## Data Availability

The original contributions presented in this study are included in the article.
